# Associations of vegetable and fruit intake with cognitive function and its decline: Two longitudinal studies

**DOI:** 10.1016/j.jnha.2024.100223

**Published:** 2024-04-09

**Authors:** Liyan Huang, Caifeng Zhao, Mengyan Gao, Yang Tao, Xiao Chen, Hui Chen, Fengping Li, Ying Zheng, Mengxi Lu, Yuan Ma, Shuang Rong, Changzheng Yuan

**Affiliations:** a School of Public Health, The Second Affiliated Hospital, Zhejiang University School of Medicine, Hangzhou, Zhejiang 30058, China; b Department of Nutrition Hygiene and Toxicology, Academy of Nutrition and Health, School of Public Health, Medical College, Wuhan University of Science and Technology, Wuhan, Hubei 430065, China; c Department of Epidemiology, Harvard T.H. Chan School of Public Health, Boston, MA 02115, United States; d School of Public Health, Wuhan University, Wuhan, Hubei 430071, China; e Department of Nutrition, Harvard T.H. Chan School of Public Health, Boston, MA 02115, United States

**Keywords:** Vegetables, Fruits, Cognitive function, Cognitive decline, Cohort study

## Abstract

**Objectives:**

Previous studies suggested protective associations of vegetables and fruits (VF) intake with cognitive function, but evidence on specific types of VF was insufficient.

**Methods:**

The current study included 4066 participants from 1997 to 2006 in the China Health and Nutrition Survey (CHNS) and 6170 participants from 2013 to 2020 in the Health and Retirement Study (HRS). Dietary intake (using 3-day 24-h dietary recalls in CHNS and food frequency questionnaire in HRS) and cognitive function (using the Telephone Interview for Cognitive Status-Modified, TICS-m) were measured. Linear mixed-effects models were used to estimate the beta coefficients (β) and the 95% confidence intervals (CI) to evaluate the association of VF with cognitive function (z-score) and its decline.

**Results:**

Highest intake of total VF was associated with better cognitive function and slower cognitive decline. Differences in cognitive function z-score between the highest and lowest tertiles of VF consumption were 0.039 (95% CI: 0.002, 0.076) for CHNS and 0.063 (95% CI: 0.026, 0.100) for HRS. The corresponding differences in annual cognitive decline were 0.011 (95% CI: 0.002, 0.021) and 0.012 (95% CI: 0.003, 0.020) units respectively. Vegetables and fruits showed independent associations with cognitive function and its decline. In specific VF subgroups, when comparing the highest to the lowest tertile intake, cruciferous vegetables (*β* = 0.058, 95% CI: 0.017, 0.100 in CHNS and *β* = 0.067, 95% CI: 0.032, 0.101 in HRS) and green leafy vegetables (*β* = 0.036, 95% CI: −0.001, 0.073 in CHNS and *β* = 0.082, 95% CI: 0.046, 0.117 in HRS) was associated with better cognitive function in both cohorts. Similarly, higher intake of dark-colored vegetables (*β* = 0.019, 95% CI: 0.008, 0.030 for red/yellow vegetables in CHNS and *β* = 0.004, 95% CI: 0.001, 0.007 for green leafy vegetables in HRS) were associated with slower cognitive decline in subsequent years. Moreover, rigorous sensitivity analyses confirmed the stability of the results.

**Conclusions:**

Our findings support the potential beneficial roles of VF, especially cruciferous vegetables, green leafy vegetables, and red/yellow vegetables, in maintaining cognitive function and slowing cognitive decline in middle-aged and older adults.

## 1 Introduction

Global aging has posed increased burdens of dementia [1] on the health care system and society [2]. Due to the challenge of reversing the progressive course of dementia, the identification of modifiable lifestyle factors to prevent age-related cognitive decline has been prioritized [3].

Dietary intake of vegetables and fruits (VF) has been associated with better cognitive outcomes in previous studies [[4], [5], [6], [7]]. A meta-analysis of six cohort studies reported that the highest intake of fruits and vegetables was associated with a 26% reduced likelihood of cognitive disorders than the lowest [8]. However, current studies mainly focus on total VF intake, with limited evidence on specific types of vegetables and fruits. In addition, studies providing comparisons across diverse populations and dietary cultures remain scarce. Although evidence was inconsistent across studies, several prospective cohort studies supported the potential long-term cognitive benefits of dark-green and deep-yellow vegetables, cruciferous vegetables, berries, citrus, and avocado among adults [6,[9], [10], [11]]. A recent review of 9 short-term (range: 5 weeks–52 weeks) randomized trials also summarized the protective effects of VF intervention, including fruit juice, avocado, and high-flavonoid VF, on cognitive health in Western populations [12].

Nevertheless, population-based evidence on the long-term cognitive role of specific vegetables and fruits is insufficient. Therefore, we aimed to investigate the associations of total and specific subgroups of VF with cognitive function and cognitive decline in two nationally representative studies of Chinese and US middle-aged and older adults.

## 2 Methods

### 2.1 Study population

We used longitudinal data from two existing community-based nationally representative cohort studies, the China Health and Nutrition Survey (CHNS) in China and the Health and Retirement Study (HRS) in the US. Detailed descriptions of these two cohorts have been published elsewhere [13,14]. Briefly, the CHNS and HRS randomly sampled nationally representative community-dwelling adults aged 50 years and over in China and the USA, respectively. The CHNS began in 1989 and follow-ups were conducted every 2–4 years. Cognitive function was assessed in 1997, 2000, 2004, and 2006 besides the regular collection of diet information. We initially included participants who completed cognitive function test at any of the four waves. Then we excluded 71 participants aged under 55 years old, 26 participants without dietary intake data, and 502 participants with baseline global cognitive scores less than 7. Finally, 4066 participants with baseline dietary assessment and at least one cognitive test were included, among whom 1357 were enrolled in 1997, 806 in 2000, 1207 in 2004, and 696 in 2006 (eFig. 1). The year when the participants were first recruited in this study was considered as baseline. In addition, the HRS commenced in 1992 and followed participants biennially until 2020. In 2013, dietary consumption was assessed in the HRS Health Care and Nutrition study (HCNS). Thus, participants who completed HCNS were initially included, and those who were younger than 55 years old (n = 1015), without any cognitive function information (n = 771), and with severe cognitive dysfunction (n = 79) were excluded. Finally, a total of 6170 participants were included in the current study (eFig. 1). The protocols of CHNS were approved by the University of North Carolina at Chapel Hill, the National Institute of Nutrition and Food Safety, and Chinese Center for Disease Control and Prevention. The HRS was approved by the Institutional Reviewing Board in University of Michigan and the National Institute on Aging. All participants provided written informed consent.

### 2.2 Vegetables and fruits

In CHNS, dietary information was collected based on validated 3-day 24-h dietary recalls at each wave. Based on 1991, 2002, and 2004 editions of the Chinese Food Composition Table, intakes of VF were calculated by combining the amounts of each food category with the unit being grams. In this study, we use the repeated assessments of dietary intake from 1997 to 2006 (eFig. 2). While in HRS, dietary intake was assessed using a validated 163-item semi-quantitative Harvard Food Frequency Questionnaire (FFQ) in 2023 (eFig. 2). Average dietary intake was calculated based on the frequency and portion size (servings) of consumption. In both cohorts, subgroups of VF included green leafy vegetables, red/yellow vegetables, cruciferous vegetables, starchy vegetables, fresh beans, other fresh vegetables, citrus fruits, berries, and other fresh fruits. Dietary intake was categorized into tertiles (low/medium/high). With the fact that there is an appreciable number of non-consumers for specific vegetable and fruit subgroups in CHNS, thus, for vegetable subgroups, we assigned non-consumers to the first tertile (low) and divided the remaining participants into medium or high groups. In terms of fruit subgroups, we divided participants dichotomously into two groups based on whether they consumed fruits or not. In order to compare consistently, we also divided participants in HRS into two groups according to whether they consumed specific fruit subgroups less than 1 serving/week. The sample size and median (interquartile range, IQR) daily intake for each group could be found in eTables 1–2. In addition, average total energy intake and major food groups were also calculated.

### 2.3 Cognitive function

Cognitive function in CHNS and HRS was evaluated by the Telephone Interview for Cognitive Status-Modified (TICS-m), a validated [15,16] and widely used tool for cognitive function assessment in large-scale studies among middle-aged and older adults [17,18]. Briefly, the cognitive assessment covers three specific cognitive domains: 1) memory (immediate and delayed recall of a 10-word list, a score of 1 was assigned when each word was recalled correctly; 2) attention (backward counting from 20 to 1, a score of 2 was given to those counted backward accurately in the first try, 1 for those only correct in the second try); 3) calculation (serial 7 subtraction [five times], a score of 1 was assigned to each of the five serial 7 subtractions). The global cognitive score (0–27 points) was the sum of the scores of verbal memory items, attention assessment items, and calculation items. The verbal memory score was the sum of the immediate and delayed 10-word recalls, with a range of 0–20. Since the global score emphasizes the domain of memory, and we further consider the comparability with other studies, a compo

site cognitive z-score was constructed for primary analysis by summing up the averaged z-scores of verbal memory items, attention assessment items, and calculation items. In this study, cognitive function was assessed in 1997, 2000, 2004, and 2006 in CHNS, and in 2014, 2016, 2018, and 2020 in HRS (eFig. 2).

### 2.4 Covariates

Structured questionnaires were used to collect information on sociodemographic characteristics, lifestyle variables, and chronic conditions in each wave both in CHNS and HRS. The sociodemographic characteristics included age, sex, education, and household income in both cohorts, while additionally included residence and region in CHNS and race in HRS. lifestyle factors included smoking status, drinking status, body mass index (BMI), total energy intake, and physical activity. Physical activity was calculated as metabolic equivalent (MET)-hours per week by self-reported hours spent in different occupational, household, transportation, and leisure-time activities per week in CHNS, whereas recorded as the frequency of moderate-to-vigorous physical activity in HRS. History of chronic diseases was collected by self-reported hypertension and diabetes diagnosis in both cohorts, and additionally included myocardial infarction in CHNS and stroke in HRS.

### 2.5 Statistical analysis

Baseline characteristics of participants were described for all participants and according to tertiles of total VF intake. Continuous variables were presented as means ± standard deviations (SDs) and categorical variables were presented as numbers (percentages).

Linear mixed-effects models were used to evaluate the association of VF with composite cognitive function and cognitive decline. Differences in the rate of cognitive decline associated with different VF intake levels were tested by including VF by time interaction term in the models. For CHNS, the rational of including 696 newly enrolled participants in 2006 in the cognitive decline analysis was that they could contribute to the parameter estimation of covariates in the linear mixed model with a time indicator of 0. A positive beta coefficient (β) presents higher VF intake was associated with better cognitive function and slower cognitive decline. We also investigated the association of total VF intake with global cognitive score, verbal memory score, and scores decline in the secondary analysis. Moreover, the associations between each VF subgroup and global cognitive function and cognitive decline were also estimated. Single imputation was performed for missing values of these covariates, using mean for continuous variables and mode for categorical variables. Model 1 adjusted for age, age square, sex, education (low: illiteracy/median: primary school/high: middle school and above), household income (tertiles), residence (rural/urban), and region (northern/southern) in CHNS, and adjusted for age, age square, sex, race (White or Caucasian/Black or African American), education (low: lower than high school/median: high school graduated/high: college and above), and household income (tertiles) in HRS. Model 2 additionally adjusted for smoking status (never/ever), drinking status (never/ever), BMI (normal, <24 kg/m^2^ in CHNS and <25 kg/m^2^ in HRS/overweight, 24–27.9 kg/m^2^ in CHNS and 25–29.9 kg/m^2^ in HRS/obesity, ≥28 kg/m^2^ in CHNS and ≥30 kg/m^2^ in HRS), total energy intake (continuous), and physical activity (tertiles). Model 3 additionally adjusted for intake of legumes, red meat, poultry, fish and aquatic products, and sweets (tertiles). Stratified analyses were conducted to explore the associations across subgroups defined by major covariates. The potential effect modification was tested including intakes of VF by covariate interaction term in the multivariable-adjusted models. In the sensitivity analyses, we further adjusted for chronic diseases, including hypertension, diabetes, and myocardial infarction in CHNS and hypertension, diabetes, and stroke in HRS, which may lie within the causal pathway of the association of VF intake with cognitive function and cognitive decline. Moreover, we excluded participants with extreme intake energy (<500 or >3500 kcal/day for women and <800 or >4000 kcal/day for men) and then repeated the primary analysis [19]. In addition, we developed a modifiable Mediterranean-Dietary Approaches to Stop Hypertension Intervention for Neurodegenerative Delay (MIND) Diet score by excluding the fruits and vegetables items initially contained in the dietary score [20,21], and further adjusted for this score in addition to model 2. Finally, we excluded participants who with missing value in covariates and repeated the primary analysis. Statistical analyses were performed using R 4.1.2 with the “nlme” package, and two-sided *P*-values below 0.05 were considered statistically significant.

## 3 Results

A total of 4066 participants from the CHNS with a mean follow-up of 4.1 years and 6170 participants from the HRS with an average of 5.2 years’ follow-up were included (Table 1). Among the CHNS participants, 49.5% were female, the mean age was 62.2 years, the average intakes of total VF, total vegetables, and total fruits were 369.7 g/d, 339.2 g/d, and 47.4 g/d (approximately 3.7 servings/d, 3.4 servings/d, and 0.5 serving/d, based on the criteria in the 2022 edition of the Chinese Dietary Guidelines of 100 g for each serving of vegetables or fruits), respectively. Of the HRS participants, 57.2% were female, the mean age was 69.1 years, the average intakes of total VF, total vegetables, and total fruits were 3.9 servings/d, 2.8 servings/d, and 1.9 serving/d, respectively. The average cognitive function scores were 16.0, 15.9, 14.4, and 13.5 across the follow-up period in CHNS and were 16.0, 15.9, 16.11, and 16.11 in HRS. Participants with higher intake of total VF were more likely to be higher educated, consumers of alcohol, have more physical activities, and have higher total energy intake.

**Table 1:** Population characteristics in CHNS (N = 4066) and HRS (N = 6170) by intake of total vegetables and fruits.

Variables	China Health and Nutrition Survey (N = 4066)			Health and Retirement Study (N = 6170)		
	Low	Medium	High	Low	Medium	High
N	1327	1429	1310	2036	2035	2099
Total vegetables and fruits intake, median (IQR)^a^	196.7 (150.0, 230.0)	333.3 (300.0, 366.7)	537.7 (466.7, 666.7)	1.8 (1.2, 2.3)	3.9 (3.4, 4.5)	7.3 (6.1, 9.1)
Age, y, mean ± SD	63.8 ± 7.6	61.9 ± 6.7	60.9 ± 6.3	68.4 ± 9.4)	69.6 ± 9.5	69.4 ± 10.1
Female, n (%)	708 (53.4)	688 (48.1)	617 (47.1)	1084 (53.2)	1168 (57.4)	1291 (61.5)
White/Caucasian, n (%)	–	–	–	1558 (76.5)	1609 (79.1)	1589 (75.7)
Resident in urban China, n (%)	597 (45.0)	589 (41.2)	482 (36.8)	–	–	–
Resident in Southern China, n (%)	679 (51.2)	879 (61.5)	818 (62.4)	–	–	–
Education, n (%)^b^						
Low	646 (48.7)	609 (42.6)	529 (40.4)	289 (14.2)	208 (10.2)	291 (13.9)
Median	317 (23.9)	383 (26.8)	351 (26.8)	833 (40.9)	728 (35.8)	620 (29.5)
High	364 (27.4)	437 (30.6)	430 (32.8)	914 (44.9)	1099 (54.0)	1188 (56.6)
Income, n (%)^c^						
Low	524 (39.5)	526 (36.8)	398 (30.4)	621 (30.5)	506 (24.9)	557 (26.5)
Median	476 (35.9)	510 (35.7)	429 (32.7)	768 (37.7)	718 (35.3)	759 (36.2)
High	327 (24.6)	393 (27.5)	483 (36.9)	647 (31.8)	811 (39.9)	783 (37.3)
Energy, kcal/d, mean ± SD	1901 ± 599.2	2103 ± 596.2	2346 ± 627.7	1345 ± 611.0	1736 ± 639.4	2460 ± 1351.2
Smoking status, n (%)						
Never	905 (68.2)	956 (66.9)	848 (64.7)	823 (40.4)	970 (47.7)	1010 (48.1)
Ever	422 (31.8)	473 (33.1)	462 (35.3)	1213 (59.6)	1065 (52.3)	1089 (51.9)
Drinking status, n (%)						
Never	924 (69.6)	963 (67.4)	842 (64.3)	967 (47.5)	898 (44.1)	880 (41.9)
Ever	403 (30.4)	466 (32.6)	468 (35.7)	1069 (52.5)	1137 (55.9)	1219 (58.1)
Physical activity, n (%)^d^						
Low	581 (43.8)	581 (40.7)	429 (32.7)	1260 (61.9)	1062 (52.2)	971 (46.3)
Median	399 (30.1)	423 (29.6)	374 (28.5)	399 (19.6)	462 (22.7)	451 (21.5)
High	347 (26.1)	425 (29.7)	507 (38.7)	377 (18.5)	511 (25.1)	677 (32.3)
BMI, n (%)^e^						
Normal	846 (63.8)	917 (64.2)	816 (62.3)	542 (26.6)	531 (26.1)	596 (28.4)
Overweight	365 (27.5)	377 (26.4)	372 (28.4)	733 (36.0)	775 (38.1)	772 (36.8)
Obesity	116 (8.7)	135 (9.4)	122 (9.3)	761 (37.4)	729 (35.8)	731 (34.8)
Hypertension, yes, n (%)	224 (16.9)	207 (14.5)	217 (16.6)	1289 (63.3)	1266 (62.2)	1233 (58.7)
Diabetes, yes, n (%)	56 (4.2)	41 (2.9)	38 (2.9)	511 (25.1)	464 (22.8)	495 (23.6)
Myocardial infarction, yes, n (%)	14 (1.1)	17 (1.2)	11 (0.8)	–	–	–
Stroke, yes, n (%)	–	–	–	188 (9.2)	137 (6.7)	162 (7.7)

*Notes*: IQR = interquartile range; BMI = body mass index.

a The units were grams/day in CHNS and servings/day in HRS.

b Education level was classified as illiteracy (low), primary school (median), and middle school and above (high) in CHNS, and lower than high school (low), high school graduated (median), and college and above (high) in HRS.

c Household income categories were defined by tertile.

d Physical activity categories were defined by tertile of metabolic equivalent (MET)-hours per week in CHNS, and were classified as never (low), 1–6 times per week (median), and every day (high) according to the exercise frequency in HRS.

e Overweight was defined as BMI being 24–27.9 kg/m^2^ in CHNS and 25–29.9 kg/m^2^ in HRS, and obesity was defined as BMI being ≥28 kg/m^2^ in CHNS and ≥30 kg/m^2^ in HRS.

**Fig. 1:**
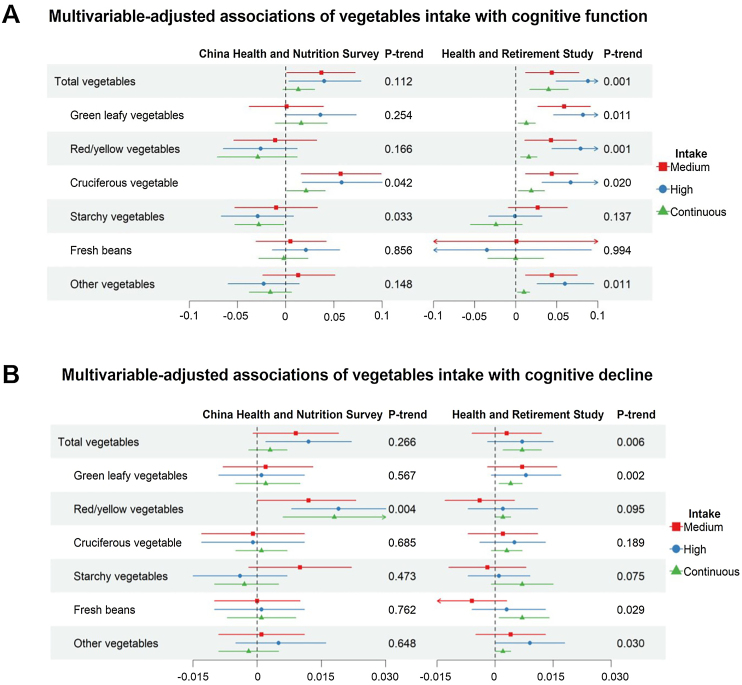
Multivariable-adjusted associations of total and specific vegetables intake with cognitive function and cognitive decline. *Notes:* Models were adjusted for age, age square, sex, education (illiteracy/primary school/middle school and above), residence (urban/rural), region (northern/southern), income (low/medium/high), smoking status (never/ever), drinking status (never/ever), BMI (normal/overweight/obesity), total intake of energy (continuous), physical activities (low/medium/high), tertiles of (low/medium/high) intake of fruits, legumes, red meat, poultry, fish and aquatic products, sweets, and mutually adjusted for tertiles of (low/medium/high) intake of green leafy vegetables, red/yellow vegetables, cruciferous vegetables, starchy vegetables, fresh beans, other vegetables in CHNS, and were adjusted for age, age square, sex, race (White or Caucasian/Black or African American), education (lower than high school/high school graduated/college and above), income (low/medium/high), smoking status (never/ever), drinking status (never/ever), BMI (normal/overweight/obesity), total intake of energy (continuous), physical activities (low/medium/high) and tertiles (low/medium/high) of intake of citrus fruits, berries, other fruits, legumes, red meat, poultry, fish and aquatic products, sweets in HRS. When the intake of vegetables was treated as a continuous variable, each 200 grams/day increase in vegetables intake in the CHNS, each 3 servings/day increment of intake of total vegetables, or each 3 servings/week increment of intake of specific vegetable subgroups in HRS were analysed.

Higher total VF intake was significantly associated with better cognitive function and slower cognitive decline (Table 2). In CHNS, compared to participants in the bottom tertile of total VF intake, individuals in the middle tertile and top tertile had 0.046 (95% confidence intervals, CI: 0.011, 0.080) and 0.039 (95% CI: 0.002, 0.076) units higher in composite cognitive function z-score. They also had 0.016 (95% CI: 0.006, 0.025) and 0.011 (95% CI: 0.002, 0.021) units slower rate of annual composite cognitive decline, respectively. Similar pattern of associations were also observed among participants in the HRS. For individuals in the middle tertile and highest tertile of total VF intake, the differences in composite cognitive function were 0.034 (95% CI: 0.003, 0.066) and 0.063 (95% CI: 0.026, 0.100), compared to participants in the bottom tertile. They also exhibited slower rates of annual composite cognitive decline by 0.005 (95% CI: −0.003, 0.014) and 0.012 (95% CI: 0.003, 0.020) units, respectively. In addition, vegetables and fruits were independently associated with cognitive function and its decline (Table 3, Fig. 1, eTables 3–4). As for cognitive function, β_T3vsT1_ (95% CI) was 0.040 (0.003, 0.078) for total vegetables and β_Yes vs No_ (95% CI) was 0.039 (0.003, 0.074) for total fruits in CHNS. Similar associations were observed for total vegetable (β_T3 vs T1_ = 0.088, 95% CI: 0.049, 0.127), but not for total fruits (β_Yes vs No_ = 0.041, 95% CI: −0.022, 0.104) in HRS. Beneficial associations were also observed for cognitive decline, β_T3vsT1_ (95% CI) was 0.012 (0.002, 0.022) for total vegetables and β_Yes vs No_ (95% CI) was 0.019 (0.010, 0.028) for total fruits in CHNS. However, significant associations were only observed when analyzing intake of vegetables and fruits as continuous variables in HRS, with β_3 servings/week increment_ (95% CI) being 0.007 (0.002, 0.012) for total vegetables and 0.007 (0.001, 0.013) for total fruits.

**Table 2:** Associations of total vegetables and fruits intake with cognitive function and cognitive decline.

	Intake of total vegetables and fruits				
	Low	Medium	High	Continuous^d^	P-trend
**China Health and Nutrition Survey (CHNS)**					
N	1327	1429	1310	4066	
Intake, g/d, median (IQR)	196.7 (150.0, 230.0)	333.3 (300.0, 366.7)	537.7 (466.7, 666.7)	333.3 (233.3, 458.3)	
**Cognitive function**					
Model 1^a^	Ref	0.074 (0.040, 0.109)	0.078 (0.043, 0.113)	0.024 (0.011, 0.038)	<0.001
Model 2^b^	Ref	0.055 (0.020, 0.089)	0.037 (0.000, 0.073)	0.008 (−0.006, 0.022)	0.249
Model 3^c^	Ref	0.046 (0.011, 0.080)	0.039 (0.002, 0.076)	0.010 (−0.004, 0.024)	0.170
**Cognitive decline**					
Model 1^a^	Ref	0.018 (0.009, 0.028)	0.017 (0.007, 0.027)	0.005 (0.001, 0.009)	0.009
Model 2^b^	Ref	0.017 (0.007, 0.026)	0.014 (0.004, 0.024)	0.004 (0.000, 0.008)	0.031
Model 3^c^	Ref	0.016 (0.006, 0.025)	0.011 (0.002, 0.021)	0.003 (−0.001, 0.007)	0.09
**Health and Retirement Study (HRS)**					
N	2036	2035	2099	6170	
Intake, servings/d, median (IQR)	1.8 (1.2, 2.3)	3.9 (3.4, 4.5)	7.3 (6.1, 9.1)	3.9 (2.3, 6.1)	
**Cognitive function**					
Model 1^a^	Ref	0.044 (0.014, 0.074)	0.062 (0.032, 0.093)	0.003 (−0.000, 0.007)	0.082
Model 2^b^	Ref	0.047 (0.016, 0.077)	0.077 (0.043, 0.111)	0.006 (0.002, 0.011)	0.006
Model 3^c^	Ref	0.034 (0.003, 0.066)	0.063 (0.026, 0.100)	0.005 (0.000, 0.010)	0.039
**Cognitive decline**					
Model 1^a^	Ref	0.005 (−0.003, 0.014)	0.011 (0.003, 0.020)	0.002 (0.000, 0.003)	0.004
Model 2^b^	Ref	0.005 (−0.003, 0.014)	0.012 (0.003, 0.020)	0.002 (0.000, 0.003)	0.004
Model 3^c^	Ref	0.005 (−0.003, 0.014)	0.012 (0.003, 0.020)	0.002 (0.000, 0.003)	0.004

*Notes*: IQR = interquartile range.

a Model 1 adjusted for age, age square, sex, education (low/medium/high), residence (urban/rural, only in CHNS), region (northern/southern, only in CHNS), race (White or Caucasian/Black or African American, only in HRS), income (low/medium/high).

b Model 2 additionally adjusted for smoking status (never/ever), drinking status (never/ever), BMI (normal/overweight/obesity), total intake of energy (continuous), and physical activities (low/medium/high).

c Model 3 additionally adjusted for tertiles (low/medium/high) of intake of legumes, red meat, poultry, fish and aquatic products, sweets.

d When the total intake of vegetables and fruits were treated as continuous variables, we estimated the difference in cognitive function z-scores for each 200 g/day increment of total vegetables and fruits intake in CHNS, and each 1 serving/day increment in HRS.

**Table 3:** Multivariable-adjusted associations of total and specific fruits intake with cognitive function and cognitive decline.

	China Health and Nutrition Survey		Health and Retirement Study	
	No	Yes	No	Yes
**Cognitive function**				
Total fruits	Ref	0.039 (0.003, 0.074)	Ref	0.041 (−0.022, 0.104)
Citrus fruits	Ref	−0.008 (−0.078, 0.062)	Ref	0.017 (−0.010, 0.043)
Berry	Ref	−0.021 (−0.117, 0.075)	Ref	−0.006 (−0.032, 0.021)
Other fruits	Ref	0.046 (0.009, 0.082)	Ref	0.049 (0.003, 0.094)
**Cognitive decline**				
Total fruits	Ref	0.019 (0.010, 0.028)	Ref	−0.001 (−0.019, 0.017)
Citrus fruits	Ref	0.006 (−0.016, 0.028)	Ref	−0.003 (−0.010, 0.005)
Berry	Ref	0.008 (−0.027, 0.044)	Ref	0.005 (−0.002, 0.012)
Other fruits	Ref	0.019 (0.010, 0.029)	Ref	0.009 (−0.004, 0.021)

*Notes*: Models were adjusted for age, age square, sex, education (illiteracy/primary school/middle school and above), residence (urban/rural), region (northern/southern), income (low/medium/high), smoking status (never/ever), drinking status (never/ever), BMI (normal/overweight/obesity), total intake of energy (continuous), physical activities (low/medium/high), tertiles of (low/medium/high) intake of vegetables, legumes, red meat, poultry, fish and aquatic products, sweets, and mutually adjusted for tertiles of (low/medium/high) intake of critus fruits, berry fruits and other fruits in CHNS, and were adjusted for age, age square, sex, race (White or Caucasian/Black or African American), education (lower than high school/high school graduated/college and above), income (low/medium/high), smoking status (never/ever), drinking status (never/ever), BMI (normal/overweight/obesity), total intake of energy (continuous), physical activities (low/medium/high) and tertiles (low/medium/high) of intake of green leafy vegetables, red/yellow vegetables, cruciferous vegetables, starchy vegetables, fresh beans, other vegetables, legumes, red meat, poultry, fish and aquatic products, sweets in HRS.

In terms of specific vegetables, highest intake of cruciferous vegetables (*β* = 0.058, 95% CI: 0.017, 0.100 in CHNS and *β* = 0.067, 95% CI: 0.032, 0.101 in HRS) and green leafy vegetables (*β* = 0.036, 95% CI: −0.001, 0.073 in CHNS and *β* = 0.082, 95% CI: 0.046, 0.117 in HRS) demonstrated positive associations with composite cognitive function in both cohorts. However, beneficial associations between intake of red/yellow vegetables (*β* = 0.079, 95% CI: 0.044, 0.114) and other vegetables (*β* = 0.060, 95% CI: 0.026, 0.095) were mainly observed in HRS. Furthermore, for cognitive decline, the association for red/yellow vegetables (*β* = 0.019, 95% CI: 0.008, 0.030) was significant in the CHNS. In HRS, green leafy vegetables (β_per 3 servings/d_ = 0.004, 95% CI: 0.001, 0.007), fresh beans (β_per 3 servings/d_ = 0.007, 95% CI: 0.001, 0.014/year), and other vegetables (β_per 3 servings/d_ = 0.002, 95% CI: 0.000, 0.004) demonstrate protective trend with cognitive decline (all P-trend <0.05) (Fig. 1, eTable 3). As for specific fruits, significant protective associations were observed for other fruits (except citrus fruits and berries) in CHNS. Participants with other fruits intake had 0.046 (0.009, 0.082) units higher composite cognitive function and 0.019 (0.010, 0.029) units slower of annual composite cognitive decline (Table 3). Non-significant associations were observed for starchy vegetables, citrus fruits, and berries in both cohorts (Table 3, Fig. 1, eTables 3–4).

In the stratified analyses, notably, the association of intake of total VF with cognitive function was stronger among individuals with normal BMI, living in rural areas, and residents in southern China in CHNS, and among participants with under high school education level in HRS (all P-interaction <0.05) (eFigs. 3–4). In sensitivity analyses (eTable 5), the results remained similar when the models were additionally adjusted for chronic diseases, and when the analyses were restricted to participants with reliable energy intake or possessed comprehensive data on covariates, and when adjusted for the modifiable MIND score. Moreover, similar associations were observed for global cognitive function z-scores, and verbal memory subdomain (eTable 6).

## 4 Discussion

In these two community-based cohort studies of Chinese and US adults aged 55 years and older, higher intake of VF were associated with better cognitive function and slower cognitive decline during an average of 4.1–5.2 years of follow-up. The differences in cognitive function z-score between the highest and lowest tertiles of VF consumption were 0.039 for CHNS and 0.063 for HRS. Corresponding differences in annual cognitive decline rate were 0.011 and 0.012 units respectively. Considering the cumulative effects of dietary consumptions on cognition, the long-term benefit from higher consumption of VF in slowing cognitive decline may eventually evolve into subsequent lower risk of dementia [22]. Furthermore, higher intake of specific VF subgroups, including cruciferous vegetables, green leafy vegetables, and red/yellow vegetables, demonstrated beneficial associations with higher cognitive function and slower annual cognitive decline. Our study had important implications for dietary interventions to prevent cognitive decline in public health and clinical practice.

Previous studies have mainly focused on the investigation of total VF consumption in relation to cognitive disorders. A recent meta-analysis of 9 observational studies (five cohort studies and four cross-sectional studies) showed a 20% lower odds of developing cognitive impairment and dementia in participants with higher consumption of VF [23]. A recent study reported that higher consumption of VF (median intake 520 g/day vs 165 g/day) was associated with a 17% lower risk of cognitive impairment among 16.7 thousand Singapore Chinese [24]. Among 18000 Hong Kong residents, adequate VF consumption (≥3 servings/d for vegetables and ≥2 servings/d for fruits) was related to 25% lower dementia risk, compared with inadequate consumption [25]. In another study conducted among US population found that high vegetable but not fruit consumption was associated with slower rate of cognitive decline with older age [5]. Moreover, in a previous cohort study in Shanghai, higher VF intake was associated with higher scores in verbal recall, digit span, and verbal fluency [26]. Similar to previous studies, our findings support the potential benefits of VF for maintaining cognitive function and slowing cognitive decline in later life. In addition, we observed stronger associations of VF among participants resident in Southern China than those in the northern area, which might be attributed to regional differences in lifestyle and cooking methods. In a previous investigation, southeast Chinese are habituated to eating more vegetables, fruits, fish, and shrimp, and prefer cooking by steaming or boiling with plant oil and less salt [27]. Nevertheless, future large-scale studies are needed to confirm the study findings and to understand the potential mechanisms.

In terms of specific VF, current evidence was from western populations. In 27,842 US male health professionals, higher consumption of green leafy vegetables, carotenoid-rich vegetables, berries, and orange juice were significantly associated with lower odds of subjective cognitive decline in late life [9]. Similarly, in 15,080 US female participants in the Nurses’ Health Study, higher green leafy vegetables and cruciferous vegetables intake were also related to slower objective cognitive decline [11]. In addition, previous studies also highlight the potential cognitive protected effect of carotenoid-rich vegetables/fruits and cruciferous vegetables [5,6]. In 3231 younger adults, tomatoes, dark-green and deep-yellow vegetables, avocado, and guacamole but not potatoes were associated with better cognitive performance in the subsequent 25 years [10]. Moreover, the beneficial effect of cabbage and root vegetables for slower cognitive decline was also reported in 2613 middle-aged population [7]. In the Chinese population, a cross-sectional study also revealed that participants who consumed green vegetables every day had significantly lower odds of mild cognitive impairment, compared with less consumers [28]. Our studies provided further evidence that higher intake of cruciferous, carotenoid-rich green leafy, red, and yellow vegetables was associated with better cognitive function, and suggested that higher intake of carotenoid-rich vegetables was associated with slower cognitive decline. However, certain inconsistent results were also observed between the two cohorts. Beneficial association between intake of other vegetables and cognitive function, as well as associations of intake of fresh beans, green leafy vegetables, and other vegetables with cognitive decline were mainly observed in HRS but not CHNS. Discrepancy of the above findings might be explained partly by the varying dietary cultures. The plant-based tradition tends to lead Chinese people to eat more vegetables with variated types than Western people, thus the long-term dietary habit might maintain a less varied vegetable consumption level in Chinese people. In addition, people in western countries have a higher intake of raw vegetables, whereas Chinese population usually eat boiled and fried vegetables. Different cooking methods might influence the nutrients content and antioxidant activity levels [29], thus affecting the beneficial effect of vegetables.

Although the underlying mechanism remains unclear and complex, several possible pathways could explain this association. Some fresh VFs are rich in anti-inflammatory or antioxidation nutrients, such as carotenoids, flavonoids, polyphenols, vitamins A, B, C, and E [[30], [31], [32], [33]]. These substances could potentially improve the metabolic milieu and reduce the brain’s oxidative stress, and suppress neuroinflammatory processes. Moreover, dysbiosis might be involved in the pathogenesis of dementia through the microbiota–gut–brain axis [34,35]. And a diet rich in VF was related to more diversified gut microbiota, which plays a protective role in maintaining cognitive health.

Our study included two nationally representative prospective cohort studies that enabled cross-national comparisons. Strengths of the study included the cross-national comparison based on two nationally representative prospective cohort studies, repeated measurements of dietary and cognitive information, long-term follow-up, and careful control of various potential confounders. However, several limitations should be cautiously accounted for when interpreting our findings. Firstly, there is heterogeneity in study design between CHNS and HRS. However, although utilization of diverse methods to assess the intake of fruits and vegetables (3-day 24-h dietary recalls in CHNS and food frequency questionnaire in HRS), along with different follow-up durations (1.1 years difference) and diverse distribution of education levels in these two cohorts, consistent results were observed. Secondly, in CHNS the 24-h dietary recalls may not fully represent long-term dietary habits, especially considering seasonal changes in VF consumption. Thirdly, both 3-day 24-h dietary recalls and food frequency questionnaires, along with covariates including physical activities and chronic diseases, rely on self-reported data, which can potentially lead to recall and reporting biases. Fourthly, the assessment of cognitive function covers only several domains of cognition, therefore, the observed associations were limited to the tested domains (memory, attention, and calculation). In addition, residual confounding and reverse causation could still exist. Finally, the observed associations are based on Chinese and US middle-aged and older adults, and the generalizability warrants further investigation.

## 5 Conclusion

In summary, our findings support the potential protective roles of vegetables and fruits in maintaining cognitive function and slowing cognitive decline among Chinese and US middle-aged and older adults. In particular, cruciferous vegetables, green leafy vegetables, and red/yellow vegetables may be the major contributors. This topic could help shed light on the association of specific VF with cognitive health and expand the existing study scope to the possibly largest aging society in the world, which is crucial to understanding how diet could play a role in age-related cognitive function among the population with different dietary cultures. Future research is warranted to confirm our findings and to investigate the underlying mechanisms.

## Authors’ contributions

CY, SR, LH, and CZ designed research; CY and LH acquired the data; CZ and LH performed the statistical analysis and interpreted the data; YT, MG and HC provided statistical support; LH and CZ developed the draft of the manuscript. CY, SR, MG, YT, XC, HC, FL, YZ, ML, and YM critically revised the manuscript for important intellectual content. CY had primary responsibility for the final content. All authors read and approved the final manuscript.

## Conflicts of interest and source of funding

This work was supported by the 10.13039/501100001809National Natural Science Foundation of China (No. 8210120183). No potential conflict of interest was reported by the authors.

## Data sharing statement

The datasets, and study materials that support the findings of our study can be found on the China Health and Nutrition Survey (CHNS) official website (http://www.cpc.unc.edu/projects/china) and the Health and Retirement Study (HRS) data products website (https://hrs.isr.umich.edu/data-products).
