# Supplementary material for: Associations of vegetable and fruit intake with cognitive function and its decline: Two longitudinal studies
Source: J Nutr Health Aging. 2024 Apr 9;28(6):100223. doi: 10.1016/j.jnha.2024.100223 (PMC12275677; doi:10.1016/j.jnha.2024.100223)
Supplement: Supplementary file 1 [file mmc1.docx]

**Title:** Associations of vegetables and fruits intake with cognitive function and its decline: two longitudinal studies

**Legends of supplementary tables and figures**

**eTable 1** Sample size and daily intake (median [IQR]) across different intake levels of total and specific vegetable subgroups

**eTable 2** Sample size and daily intake (median [IQR]) across different intake levels of total and specific fruit subgroups

**eTable 3** Multivariable-adjusted associations of total and specific vegetables intake with cognitive function and cognitive decline

**eTable 4** Multivariable-adjusted associations of fruits intake with global cognitive function and global cognitive decline in HRS

**eTable 5** Sensitivity analysis for associations of total vegetables and fruits intake with cognitive function and cognitive decline

**eTable 6** Associations of total vegetables and fruits intake with global cognitive z-score and verbal memory z-score

**eFigure 1** Flowchart of participants inclusion

**eFigure 2** Temporal sequence of the measurements of diet and cognitive function used in the study

**eFigure 3** Multivariable-adjusted associations of total vegetables and fruits intake (T3 VS. T1) with cognitive function and cognitive decline in subgroups in CHNS

**eFigure 4** Multivariable-adjusted associations of total vegetables and fruits intake (T3 VS. T1) with cognitive function and cognitive decline in subgroups in HRS

| **eTable 1** Sample size and daily intake (median [IQR]) across different intake levels of total and specific vegetable groups | | | | |
| --- | --- | --- | --- | --- |
| Food Group | Foods examples | Intake levels | | |
|  |  | No/ Low | Medium | High |
| **China Health and Nutrition Survey** | | | | |
| Total vegetables |  | 1131 ^a^ | 1452 | 1483 |
|  |  | 166.7 (132.1, 200.0) ^b^ | 300.0 (265.0, 325.0) | 468.3 (403.3, 583.3) |
| Green leafy vegetables | Spinach, water spinach, lettuce, collards, mustard leaf, pak choi, etc. | 2470 | 764 | 832 |
|  |  | 0.0 (0.0, 0.0) | 100.0 (50.0, 100.0) | 200.0 (160.0, 266.7) |
| Red/yellow vegetables | Carrot, tomato, red spinach, color pepper, etc. | 2912 | 523 | 631 |
|  |  | 0.0 (0.0, 0.0) | 50.0 (30.0, 70.0) | 133.3 (100.0, 200.0) |
| Cruciferous vegetable | Chinese cabbage, broccoli, senvy, turnip, etc. | 620 | 1688 | 1758 |
|  |  | 0.0 (0.0, 0.0) | 125.0 (100.0, 150.0) | 271.7 (225.0, 351.9) |
| Starchy vegetables | Taro, lotus root, Chinese yam, etc. | 2888 | 517 | 661 |
|  |  | 0.0 (0.0, 0.0) | 100.0 (65.0, 100.0) | 200.0 (175.0, 285.0) |
| Fresh beans | Bean sprout, cowpea, kidney bean, pea seedlings, etc. | 2255 | 892 | 919 |
|  |  | 0.0 (0.0, 0.0) | 100.0 (60.0, 100.0) | 200.0 (166.7, 262.5) |
| Other vegetables | Cucumber, eggplant, garlic, onion, bamboo shoots, etc. | 1035 | 1588 | 1443 |
|  |  | 0.0 (0.0, 0.0) | 75.0 (50.0, 100.0) | 200.0 (160.0, 275.0) |
| **Health and Retirement Study** | | | | |
| Total vegetables |  | 2031 | 2040 | 2099 |
|  |  | 0.9 (0.6, 1.2) ^c^ | 2.1 (1.8, 2.6) | 4.4 (3.6, 5.8) |
| Green leafy vegetables | Spinach, kale, lettuce | 1874 | 2159 | 2137 |
|  |  | 0.6 (0.1, 1.0) ^d^ | 2.0 (1.5, 2.5) | 6.5 (4.5, 8.7) |
| Red/yellow vegetables | Tomatoes, tomato juice, tomato sauce, carrots, squash | 2033 | 1988 | 2149 |
|  |  | 1.1 (0.7, 1.6) | 3.6 (3.0, 4.5) | 8.1 (6.7, 11.0) |
| Cruciferous vegetable | Broccoli, cauliflower, cabbage, sauerkraut, brussels sprouts | 1936 | 2046 | 2188 |
|  |  | 0.2 (0.1, 0.6) | 1.1 (1.0, 1.5) | 3.5 (2.1, 5.0) |
| Starchy vegetables | Corn, yams/sweet potatoes | 1738 | 1313 | 3119 |
|  |  | 0.1 (0.0, 0.5) | 0.6 (0.6, 0.6) | 1.0 (1.0, 3.0) |
| Fresh beans | Beans | 1342 | 2566 | 2262 |
|  |  | 0.1 (0.0, 0.1) | 1.0 (0.6, 1.0) | 3.0 (1.5, 4.0) |
| Other vegetables | Mixed vegetables, squash, eggplant/zucchini, green pepper, garlic, celery, mushrooms, alfalfa sprouts, beets, onion | 2020 | 2052 | 2098 |
|  |  | 1.2 (0.7, 1.7) | 4.0 (3.0, 5.2) | 11.1 (8.6, 15.5) |
| ^a^Sample size (all such values).  ^b^Grams of daily intake (median [IQR]) (all such values in CHNS).  ^c^Servings of daily intake (median [IQR]) (all such values for total vegetables in HRS).  ^d^Servings of weekly intake (median [IQR]) (all such values for specific vegetable subgroups in HRS). | | | | |
|  | | | | |
|  | | | | |
|  | | | | |

| **eTable 2** Sample size and daily intake (median [IQR]) across different intake levels of total and specific fruit groups | | | | |
| --- | --- | --- | --- | --- |
| Food Group | Foods examples | | Intake levels | |
|  |  |  | No | Yes |
| **China Health and Nutrition Survey** | | | | |
| Total fruits |  | 3124 ^a^ | | 942 |
|  |  | 0.0 (0.0, 0.0) ^b^ | | 150.0 (100.0, 233.3) |
| Citrus fruits | Oranges, tangerines, pomelos, etc. | 3927 | | 139 |
|  |  | 0.0 (0.0, 0.0) | | 125.0 (95.0, 181.7) |
| Berries | Grapes, strawberries, blueberries, etc. | 3985 | | 81 |
|  |  | 0.0 (0.0, 0.0) | | 175.0 (100.0, 250.0) |
| Other fruits | Apple, pear, watermelon, banana, peach, etc. | 3206 | | 860 |
|  |  | 0.0 (0.0, 0.0) | | 150.0 (100.0, 225.0) |
| **Health and Retirement Study** | | | | |
| Total fruits |  | | 262 | 5908 |
|  |  |  | 0.1 (0.0, 0.1) ^c^ | 1.6 (0.9, 2.6) |
| Citrus fruits | Oranges, grapefruit, orange juice, grapefruit juice | | 2288 | 3882 |
|  |  |  | 0.1 (0.0, 0.6) ^d^ | 3.6 (1.6, 7.1) |
| Berries | Strawberries, blueberries | | 3366 | 2804 |
|  |  |  | 0.1 (0.0, 0.5) | 3.0 (1.0, 4.0) |
| Other fruits | Raisins/grapes, prunes, avocado, bananas, cantaloupe, watermelon, apples/pears, applesauce, peaches/apricots/plums, apple juice, prune juice, other juice | | 539 | 5631 |
|  |  |  | 0.5 (0.2, 0.7) | 7.2 (3.6, 11.6) |
| ^a^Sample size (all such values).  ^b^Grams of daily intake (median [IQR]) (all such values in CHNS).  ^c^Servings of daily intake (median [IQR]) (all such values for total fruits in HRS).  ^d^Servings of weekly intake (median [IQR]) (all such values for specific fruit subgroups in HRS). | | | | |
|  | | | | |
|  | | | | |
|  | | | | |

| **eTable 3** Multivariable-adjusted associations of total and specific vegetables intake with cognitive function and cognitive decline | | | | | |
| --- | --- | --- | --- | --- | --- |
|  | Intake of total and specific vegetables | | | |  |
|  | Low | Medium | High | Continuous^a^ | P-trend |
| **China Health and Nutrition Survey** | | |  |  |  |
| **Cognitive function** |  |  |  |  |  |
| Total vegetables | Ref | 0.037 (0.001, 0.072) | 0.040 (0.003, 0.078) | 0.013 (-0.003, 0.030) | 0.112 |
| Green leafy vegetables | Ref | 0.001 (-0.038, 0.039) | 0.036 (-0.001, 0.073) | 0.016 (-0.011, 0.043) | 0.254 |
| Red/yellow vegetables | Ref | -0.011 (-0.054, 0.032) | -0.026 (-0.065, 0.012) | -0.029 (-0.071, 0.012) | 0.166 |
| Cruciferous vegetable | Ref | 0.057 (0.016, 0.099) | 0.058 (0.017, 0.100) | 0.021 (0.001, 0.041) | 0.042 |
| Starchy vegetables | Ref | -0.010 (-0.053, 0.033) | -0.029 (-0.067, 0.008) | -0.028 (-0.053, -0.002) | 0.033 |
| Fresh beans | Ref | 0.005 (-0.031, 0.042) | 0.021 (-0.014, 0.056) | -0.002 (-0.028, 0.023) | 0.856 |
| Other vegetables | Ref | 0.013 (-0.024, 0.051) | -0.023 (-0.060, 0.014) | -0.016 (-0.038, 0.006) | 0.148 |
| **Cognitive decline** |  |  |  |  |  |
| Total vegetables | Ref | 0.009 (-0.001, 0.019) | 0.012 (0.002, 0.022) | 0.003 (-0.002, 0.007) | 0.266 |
| Green leafy vegetables | Ref | 0.002 (-0.008, 0.013) | 0.001 (-0.009, 0.011) | 0.002 (-0.005, 0.010) | 0.567 |
| Red/yellow vegetables | Ref | 0.012 (-0.000, 0.023) | 0.019 (0.008, 0.030) | 0.018 (0.006, 0.031) | 0.004 |
| Cruciferous vegetable | Ref | -0.001 (-0.013, 0.011) | -0.001 (-0.013, 0.011) | 0.001 (-0.005, 0.007) | 0.685 |
| Starchy vegetables | Ref | 0.010 (-0.002, 0.022) | -0.004 (-0.015, 0.007) | -0.003 (-0.010, 0.005) | 0.473 |
| Fresh beans | Ref | 0.000 (-0.010, 0.010) | 0.001 (-0.010, 0.011) | 0.001 (-0.007, 0.009) | 0.762 |
| Other vegetables | Ref | 0.001 (-0.009, 0.011) | 0.005 (-0.005, 0.016) | -0.002 (-0.009, 0.005) | 0.648 |
| **Health and Retirement Study** | | |  |  |  |
| **Cognitive function** |  |  |  |  |  |
| Total vegetables | Ref | 0.044 (0.012, 0.077) | 0.088 (0.049, 0.127) | 0.040 (0.017, 0.064) | 0.001 |
| Green leafy vegetables | Ref | 0.059 (0.027, 0.091) | 0.082 (0.046, 0.117) | 0.013 (0.003, 0.024) | 0.011 |
| Red/yellow vegetables | Ref | 0.043 (0.011, 0.074) | 0.079 (0.044, 0.114) | 0.016 (0.006, 0.026) | 0.001 |
| Cruciferous vegetable | Ref | 0.044 (0.012, 0.076) | 0.067 (0.032, 0.101) | 0.019 (0.003, 0.035) | 0.02 |
| Starchy vegetables | Ref | 0.027 (-0.009, 0.063) | -0.001 (-0.033, 0.032) | -0.024 (-0.055, 0.008) | 0.137 |
| Fresh beans | Ref | 0.001 (-0.111, 0.113) | -0.035 (-0.162, 0.092) | 0.000 (-0.034, 0.034) | 0.994 |
| Other vegetables | Ref | 0.044 (0.012, 0.075) | 0.060 (0.026, 0.095) | 0.010 (0.002, 0.017) | 0.011 |
| **Cognitive decline** |  |  |  |  |  |
| Total vegetables | Ref | 0.003 (-0.006, 0.012) | 0.007 (-0.002, 0.015) | 0.007 (0.002, 0.012) | 0.006 |
| Green leafy vegetables | Ref | 0.007 (-0.002, 0.016) | 0.008 (-0.001, 0.017) | 0.004 (0.001, 0.007) | 0.002 |
| Red/yellow vegetables | Ref | -0.004 (-0.013, 0.005) | 0.002 (-0.007, 0.011) | 0.002 (-0.000, 0.004) | 0.095 |
| Cruciferous vegetable | Ref | 0.002 (-0.007, 0.011) | 0.005 (-0.004, 0.013) | 0.003 (-0.001, 0.007) | 0.189 |
| Starchy vegetables | Ref | -0.002 (-0.012, 0.008) | 0.001 (-0.007, 0.009) | 0.007 (-0.001, 0.015) | 0.075 |
| Fresh beans | Ref | -0.006 (-0.016, 0.003) | 0.003 (-0.006, 0.013) | 0.007 (0.001, 0.014) | 0.029 |
| Other vegetables | Ref | 0.004 (-0.005, 0.013) | 0.009 (0.000, 0.018) | 0.002 (0.000, 0.004) | 0.03 |
| *Notes:* Models were adjusted for age, age square, sex, education (illiteracy/ primary school/ middle school and above), residence (urban/rural), region (northern/ southern), income (low/ medium/ high), smoking status (never/ ever), drinking status (never/ ever), BMI (normal/overweight/obesity), total intake of energy (continuous), physical activities (low/ medium/ high), tertiles of (low/ medium/ high) intake of fruits, legumes, red meat, poultry, fish and aquatic products, sweets, and mutually adjusted for tertiles of (low/ medium/ high) intake of green leafy vegetables, red/yellow vegetables, cruciferous vegetable, starchy vegetables, fresh beans, other vegetables in CHNS, and were adjusted for age, age square, sex, race (White or Caucasian/ Black or African American), education (lower than high school/ high school graduated/ college and above), income (low/ medium/ high), smoking status (never/ ever), drinking status (never/ ever), BMI (normal/overweight/obesity), total intake of energy (continuous), physical activities (low/ medium/ high) and tertiles (low/ medium/ high) of intake of citrus fruits, berries, other fruits, legumes, red meat, poultry, fish and aquatic products, sweets in HRS. | | | | | |
| ^a^When the intake of vegetables was treated as a continuous variable, each 200 grams/day increase in vegetable intake in the CHNS, each 3 servings/day increment of intake of total vegetables or each 3 servings/week increment of intake of specific vegetable subgroup in HRS were analysed. | | | | | |

| **eTable 4** Multivariable-adjusted associations of fruits intake with global cognitive function and global cognitive decline in HRS | | | | | |
| --- | --- | --- | --- | --- | --- |
|  | Tertile of intake | | |  |  |
|  | Low | Medium | High | Continuous^a^ | P-trend |
| **Cognitive function** | |  |  |  |  |
| Total fruits | Ref | 0.042 (0.011, 0.073) | -0.004 (-0.039, 0.031) | -0.025 (-0.049, 0.000) | 0.052 |
| Citrus fruits | Ref | 0.033 (0.002, 0.063) | 0.003 (-0.029, 0.035) | -0.007 (-0.015, 0.001) | 0.094 |
| Berries | Ref | 0.037 (0.003, 0.071) | 0.013 (-0.019, 0.044) | -0.008 (-0.024, 0.008) | 0.302 |
| Other fruits | Ref | 0.029 (-0.002, 0.060) | 0.015 (-0.019, 0.050) | -0.004 (-0.009, 0.002) | 0.174 |
| **Cognitive decline** | |  |  |  |  |
| Total fruits | Ref | -0.001 (-0.010, 0.007) | 0.006 (-0.002, 0.015) | 0.007 (0.001, 0.013) | 0.028 |
| Citrus fruits | Ref | -0.001 (-0.009, 0.008) | -0.000 (-0.009, 0.008) | 0.001 (-0.001, 0.003) | 0.432 |
| Berries | Ref | 0.000 (-0.010, 0.010) | 0.005 (-0.003, 0.013) | 0.001 (-0.004, 0.005) | 0.74 |
| Other fruits | Ref | 0.011 (0.003, 0.020) | 0.008 (-0.000, 0.017) | 0.002 (0.000, 0.003) | 0.008 |
| *Notes:* Models were adjusted for age, age square, sex, education (illiteracy/ primary school/ middle school and above), residence (urban/rural), region (northern/ southern), income (low/ medium/ high), smoking status (never/ ever), drinking status (never/ ever), BMI (normal/overweight/obesity), total intake of energy (continuous), physical activities (low/ medium/ high), tertiles of (low/ medium/ high) intake of vegetables, legumes, red meat, poultry, fish and aquatic products, sweets, and mutually adjusted for tertiles of (low/ medium/ high) intake of critus fruits, berry fruits and other fruits in CHNS, and were adjusted for age, age square, sex, race (White or Caucasian/ Black or African American), education (lower than high school/ high school graduated/ college and above), income (low/ medium/ high), smoking status (never/ ever), drinking status (never/ ever), BMI (normal/overweight/obesity), total intake of energy (continuous), physical activities (low/ medium/ high) and tertiles (low/ medium/ high) of intake of green leafy vegetables, red/ yellow vegetables, cruciferous vegetables, starchy vegetables, fresh beans, other vegetables, legumes, red meat, poultry, fish and aquatic products, sweets in HRS. | | | | | |
| aWhen treated as continuous variables, each 1 serving/day increment of total fruits intake, and each 3 servings/week increment of citrus fruits, berries, and other fruits were analysed. | | | | | |

| **eTable 5** Sensitivity analysis for associations of total vegetables and fruits intake with cognitive function and cognitive decline | | | | | | | |
| --- | --- | --- | --- | --- | --- | --- | --- |
|  | China Health and Nutrition Survey | | |  | Health and Retirement Study | | |
|  | Low | Medium | High |  | Low | Medium | High |
| **Cognitive function** | |  |  |  |  |  |  |
| **Additionally adjusting for chronic disease** | | | | | | | |
| N | 1327 | 1429 | 1310 |  | 2036 | 2035 | 2099 |
| Model a | Ref | 0.046 (0.011, 0.080) | 0.040 (0.004, 0.077) |  | Ref | 0.033 (0.001, 0.064) | 0.061 (0.024, 0.098) |
| **Excluding participants with extreme intake energy** | | | | | | | |
| N | 1319 | 1431 | 1299 |  | 1871 | 2002 | 1910 |
| Model b | Ref | 0.041 (0.006, 0.076) | 0.032 (-0.005, 0.069) |  | Ref | 0.032 (-0.001, 0.064) | 0.058 (0.018, 0.097) |
| **Adjusting for MIND score** | | | | | | | |
| N | 1327 | 1429 | 1310 |  | 2036 | 2035 | 2099 |
| Model c | Ref | 0.054 (0.020, 0.089) | 0.036 (-0.000, 0.073) |  | Ref | 0.045 (0.014, 0.076) | 0.074 (0.040, 0.109) |
| **Excluding participants with missing value of covariates** | | | | | | | |
| N | 1179 | 1161 | 1206 |  | 1980 | 1980 | 2041 |
| Model b | Ref | 0.029 (-0.011, 0.069) | 0.024 (-0.013, 0.062) |  | Ref | 0.031 (-0.002, 0.063) | 0.058 (0.021, 0.096) |
| **cognitive decline** | |  |  |  |  |  |  |
| **additionally adjusting for chronic disease** | | | | | | | |
| N | 1327 | 1429 | 1310 |  | 2036 | 2035 | 2099 |
| Model a | Ref | 0.016 (0.006, 0.025) | 0.011 (0.001, 0.021) |  | Ref | 0.006 (-0.003, 0.014) | 0.012 (0.003, 0.020) |
| **Excluding participants with extreme intake energy** | | | | | | | |
| N | 1319 | 1431 | 1299 |  | 1871 | 2002 | 1910 |
| Model b | Ref | 0.016 (0.006, 0.025) | 0.011 (0.001, 0.021) |  | Ref | 0.003 (-0.006, 0.012) | 0.008 (-0.001, 0.017) |
| **Adjusting for MIND score** | | | | | | | |
| N | 1327 | 1429 | 1310 |  | 2036 | 2035 | 2099 |
| Model c | Ref | 0.017 (0.007, 0.026) | 0.014 (0.004, 0.024) |  | Ref | 0.005 (-0.003, 0.014) | 0.011 (0.003, 0.020) |
| **Excluding participants with missing value of covariates** | | | | | | | |
| N | 1179 | 1161 | 1206 |  | 1980 | 1980 | 2041 |
| Model b | Ref | 0.008 (-0.003, 0.018) | 0.012 (0.002, 0.022) |  | Ref | 0.005 (-0.003, 0.014) | 0.011 (0.003, 0.020) |
| *Notes:* Model a adjusted for age, age square, sex, education (low/ medium/high), residence (urban/rural, only in CHNS), region (northern/ southern, only in CHNS), race (White or Caucasian/ Black or African American, only in HRS), income (low/ medium/ high), smoking status (never/ ever), drinking status (never/ ever), BMI (normal/ overweight/ obesity), total intake of energy (continuous), physical activities (low/ medium/ high), tertiles (low/ medium/ high) of intake of legumes, red meat, poultry, fish and aquatic products, sweets, and chronic disease of hypertension, diabetes, myocardial infarction (only in CHNS), and stroke(only in HRS). | | | | | | | |
| Model b adjusted for age, age square, sex, education (low/ medium/high), residence (urban/rural, only in CHNS), region (northern/ southern, only in CHNS), race (White or Caucasian/ Black or African American, only in HRS), income (low/ medium/ high), smoking status (never/ ever), drinking status (never/ ever), BMI (normal/ overweight/ obesity), total intake of energy (continuous), physical activities (low/ medium/ high), and tertiles (low/ medium/ high) of intake of legumes, red meat, poultry, fish and aquatic products, sweets. | | | | | | | |
| Model c adjusted for age, age square, sex, education (low/ medium/high), residence (urban/rural, only in CHNS), region (northern/ southern, only in CHNS), race (White or Caucasian/ Black or African American, only in HRS), income (low/ medium/ high), smoking status (never/ ever), drinking status (never/ ever), BMI (normal/ overweight/ obesity), total intake of energy (continuous), physical activities (low/ medium/ high), and MIND score. | | | | | | | |

| **eTable 6** Associations of total vegetables and fruits intake with global cognitive z-score and verbal memory z-score | | | | | |
| --- | --- | --- | --- | --- | --- |
|  | Intake of total vegetables and fruits | | | |  |
|  | Low | Medium | High | Continuous^a^ | P-trend |
| **China Health and Nutrition Survey** | | | | | |
| N | 1327 | 1429 | 1310 | 4066 |  |
| Intake, g/d, median (IQR) | 196.7 (150.0, 230.0) | 333.3 (300.0, 366.7) | 537.7 (466.7, 666.7) | 333.3 (233.3, 458.3) |  |
| **Cognitive function** | | | | | |
| **Global cognitive function** | |  |  |  |  |
| Model 1 | Ref | 0.083 (0.039, 0.127) | 0.089 (0.044, 0.134) | 0.030 (0.013, 0.047) | 0.001 |
| Model 2 | Ref | 0.057 (0.013, 0.101) | 0.036 (-0.011, 0.082) | 0.009 (-0.009, 0.026) | 0.334 |
| Model 3 | Ref | 0.047 (0.003, 0.092) | 0.037 (-0.010, 0.084) | 0.010 (-0.007, 0.028) | 0.254 |
| **Verbal memory scores** | | | | | |
| Model 1 | Ref | 0.072 (0.025, 0.120) | 0.080 (0.032, 0.128) | 0.028 (0.010, 0.047) | 0.002 |
| Model 2 | Ref | 0.048 (0.001, 0.095) | 0.029 (-0.020, 0.079) | 0.009 (-0.010, 0.028) | 0.361 |
| Model 3 | Ref | 0.041 (-0.007, 0.088) | 0.029 (-0.021, 0.079) | 0.010 (-0.009, 0.029) | 0.316 |
| **Cognitive decline** |  |  |  |  |  |
| **Global cognitive function** | |  |  |  |  |
| Model 1 | Ref | 0.017 (0.004, 0.029) | 0.017 (0.004, 0.030) | 0.004 (-0.001, 0.010) | 0.086 |
| Model 2 | Ref | 0.015 (0.003, 0.027) | 0.013 (0.001, 0.026) | 0.003 (-0.002, 0.008) | 0.191 |
| Model 3 | Ref | 0.013 (0.001, 0.026) | 0.011 (-0.002, 0.024) | 0.003 (-0.003, 0.008) | 0.33 |
| **Verbal memory scores** | | | | | |
| Model 1 | Ref | 0.012 (-0.001, 0.026) | 0.013 (-0.001, 0.027) | 0.002 (-0.004, 0.008) | 0.484 |
| Model 2 | Ref | 0.011 (-0.003, 0.024) | 0.010 (-0.004, 0.024) | 0.001 (-0.004, 0.007) | 0.695 |
| Model 3 | Ref | 0.009 (-0.004, 0.023) | 0.008 (-0.006, 0.022) | 0.001 (-0.005, 0.006) | 0.834 |
| **Health and Retirement Study** | | | | | |
| N | 2036 | 2035 | 2099 | 6170 |  |
| Intake, servings/d, median (IQR) | 1.8 (1.2, 2.3) | 3.9 (3.4, 4.5) | 7.3 (6.1, 9.1) | 3.9 (2.3, 6.1) |  |
| **Cognitive function** | | | | | |
| **Global cognitive function** | |  |  |  |  |
| Model 1 | Ref | 0.064 (0.023, 0.105) | 0.079 (0.038, 0.120) | 0.003 (-0.002, 0.008) | 0.206 |
| Model 2 | Ref | 0.070 (0.028, 0.112) | 0.107 (0.061, 0.153) | 0.009 (0.003, 0.015) | 0.005 |
| Model 3 | Ref | 0.053 (0.010, 0.097) | 0.086 (0.036, 0.136) | 0.008 (0.001, 0.014) | 0.028 |
| **Verbal memory scores** | | | | | |
| Model 1 | Ref | 0.059 (0.019, 0.100) | 0.091 (0.051, 0.131) | 0.007 (0.002, 0.012) | 0.004 |
| Model 2 | Ref | 0.058 (0.017, 0.099) | 0.101 (0.056, 0.146) | 0.011 (0.005, 0.017) | <0.001 |
| Model 3 | Ref | 0.045 (0.002, 0.087) | 0.082 (0.033, 0.132) | 0.009 (0.003, 0.016) | 0.005 |
| **Cognitive decline** |  |  |  |  |  |
| **Global cognitive function** | |  |  |  |  |
| Model 1 | Ref | 0.010 (-0.000, 0.021) | 0.018 (0.008, 0.029) | 0.002 (0.001, 0.004) | <0.001 |
| Model 2 | Ref | 0.011 (-0.000, 0.021) | 0.018 (0.008, 0.029) | 0.002 (0.001, 0.004) | <0.001 |
| Model 3 | Ref | 0.011 (-0.000, 0.021) | 0.018 (0.008, 0.029) | 0.002 (0.001, 0.004) | <0.001 |
| **Verbal memory scores** | | | | | |
| Model 1 | Ref | 0.011 (-0.000, 0.023) | 0.022 (0.011, 0.033) | 0.002 (0.001, 0.004) | 0.001 |
| Model 2 | Ref | 0.012 (0.000, 0.023) | 0.022 (0.011, 0.033) | 0.002 (0.001, 0.004) | 0.001 |
| Model 3 | Ref | 0.012 (0.000, 0.023) | 0.022 (0.011, 0.033) | 0.002 (0.001, 0.004) | 0.001 |
| *Notes:* Model 1 adjusted for age, age square, sex, education (low/ medium/high), residence (urban/rural, only in CHNS), region (northern/ southern, only in CHNS), race (White or Caucasian/ Black or African American, only in HRS), income (low/ medium/ high). | | | | | |
| Model 2 additionally adjusted for smoking status (never/ ever), drinking status (never/ ever), BMI (normal/ overweight/ obesity), total intake of energy (continuous), and physical activities (low/ medium/ high). | | | | | |
| Model 3 additionally adjusted for tertiles (low/ medium/ high) of intake of legumes, red meat, poultry, fish and aquatic products, sweets. | | | | | |
| ^a^When the total intake of vegetables and fruits were treated as continuous variables, each 200 grams/day increase in total vegetables and fruits intake in the CHNS and each 1 servings/day increment in HRS were analysed. | | | | | |


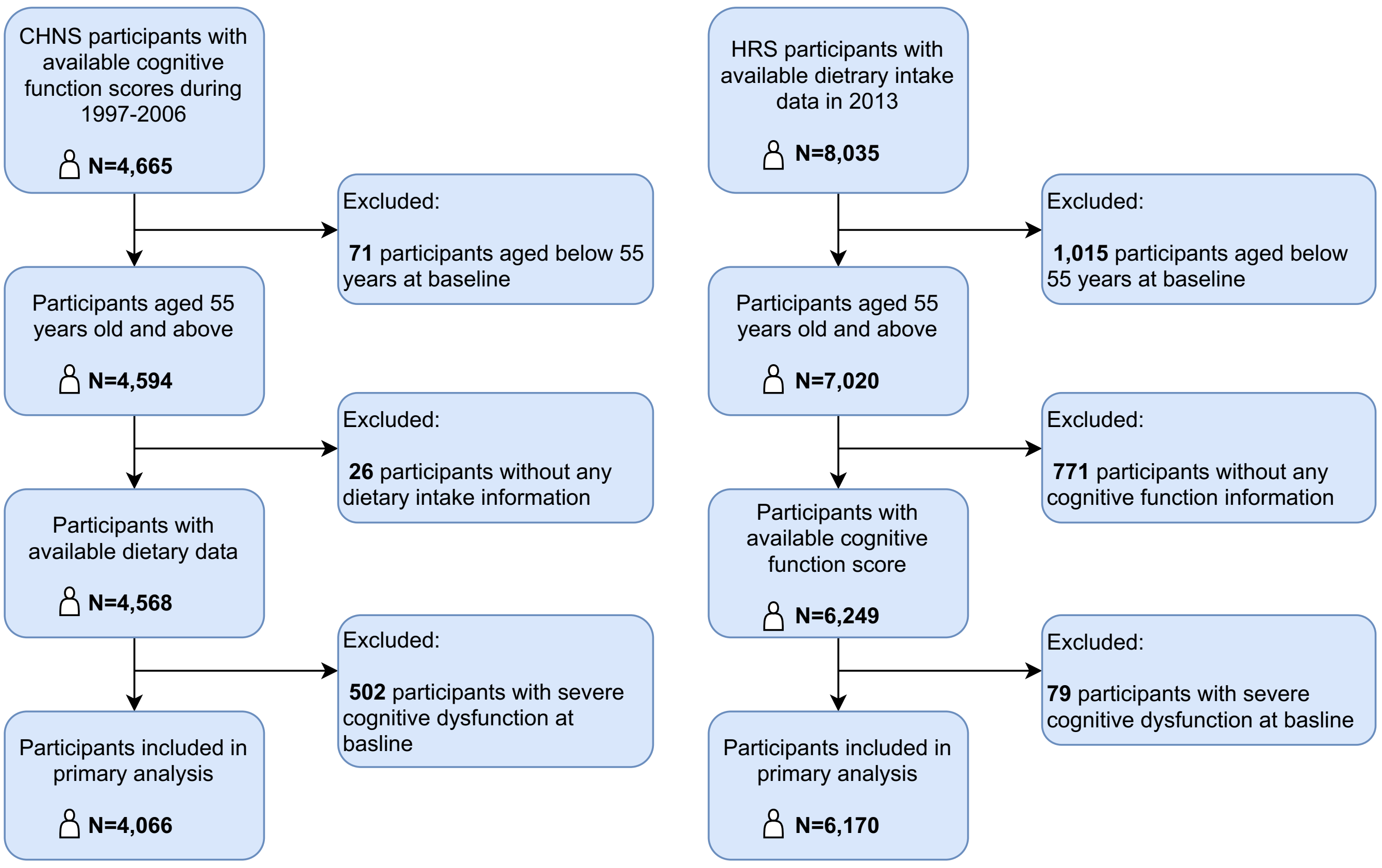


**eFigure 1.** Flowchart of participants inclusion


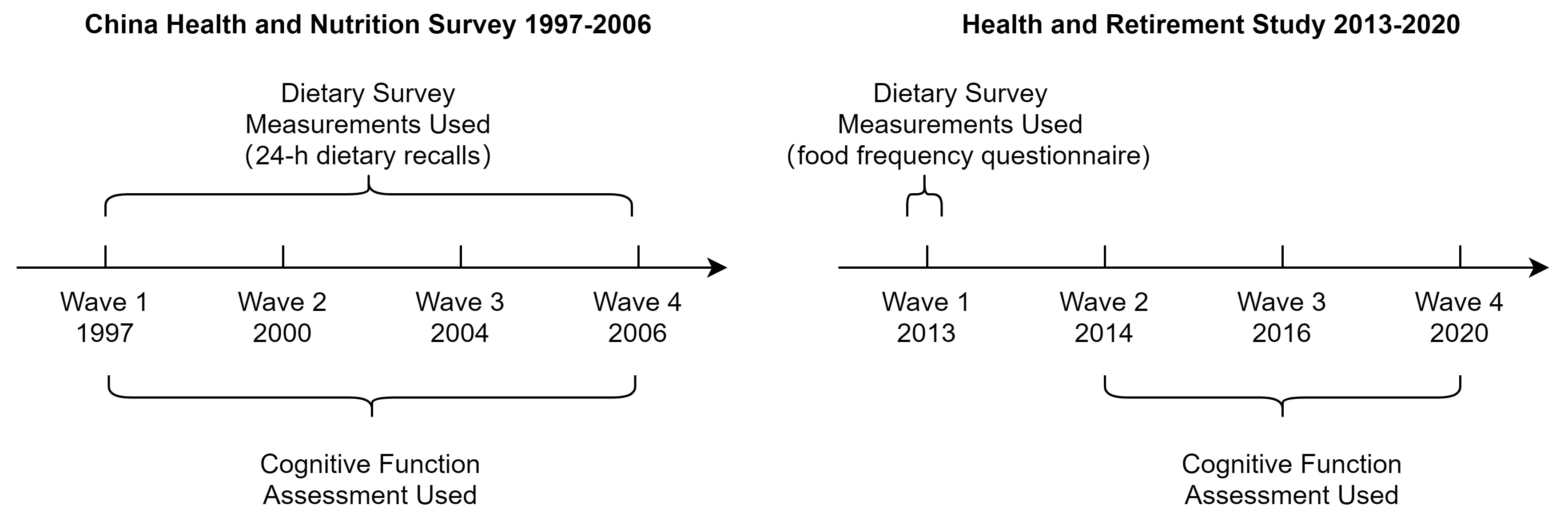


**eFigure 2** Temporal sequence of the measurements of diet and cognitive function used in the study


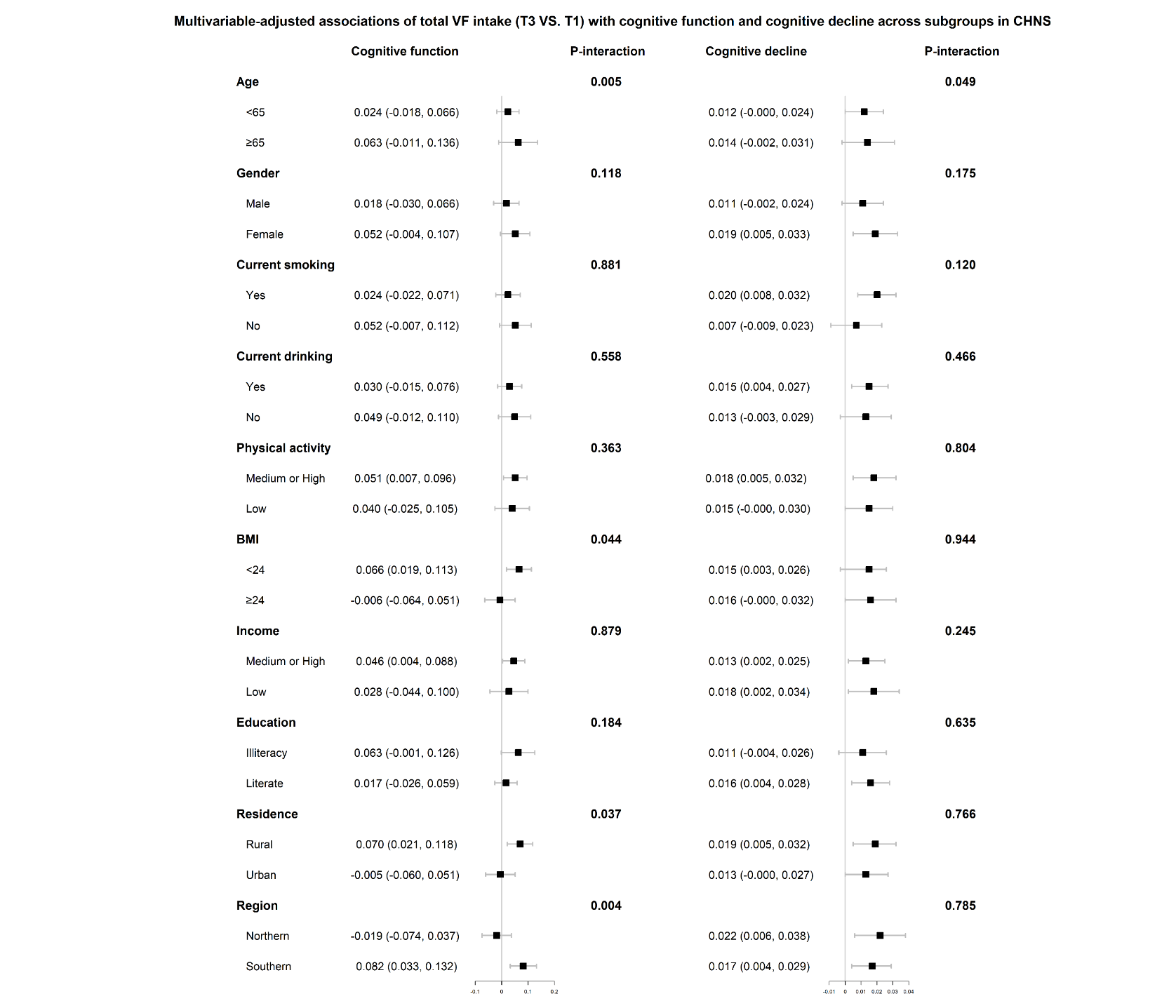


**eFigure 3** Multivariable-adjusted associations of total vegetables and fruits intake (T3 VS T1) with cognitive function and cognitive decline in subgroups in CHNS

*Notes:* Models were adjusted for age, age square, sex, education (illiteracy/ primary school/ middle school and above), residence (urban/rural), region (northern/ southern), income (low/ medium/ high), smoking status (never/ ever), drinking status (never/ ever), BMI (normal/overweight/obesity), total intake of energy (continuous), physical activities (low/ medium/ high), tertiles of (low/ medium/ high) intake of fruits, legumes, red meat, poultry, fish and aquatic products, sweets.


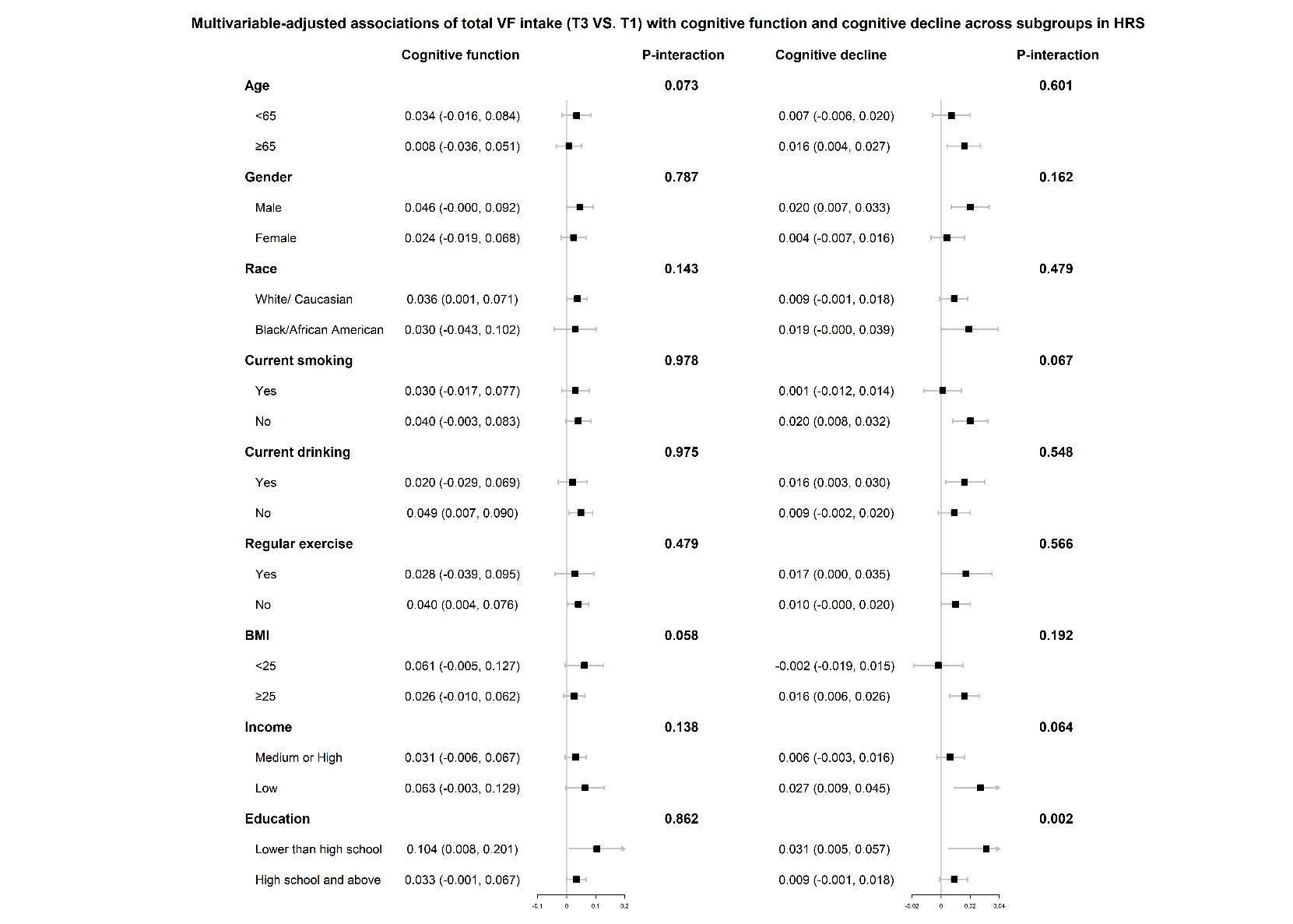


**eFigure 4** Multivariable-adjusted associations of total vegetables and fruits intake (T3 VS T1) with cognitive function and cognitive decline in subgroups in HRS

*Notes:* Models were adjusted for age, age square, sex, race (White or Caucasian/ Black or African American), education (lower than high school/ high school graduated/ college and above), income (low/ medium/ high), smoking status (never/ ever), drinking status (never/ ever), BMI (normal/overweight/obesity), total intake of energy (continuous), physical activities (low/ medium/ high) and tertiles (low/ medium/ high) of intake of citrus fruits, berries, other fruits, legumes, red meat, poultry, fish and aquatic products, sweets.
